# SARS-CoV-2 M^pro^ inhibitors with antiviral activity in a transgenic mouse model

**DOI:** 10.1126/science.abf1611

**Published:** 2021-02-18

**Authors:** Jingxin Qiao, Yue-Shan Li, Rui Zeng, Feng-Liang Liu, Rong-Hua Luo, Chong Huang, Yi-Fei Wang, Jie Zhang, Baoxue Quan, Chenjian Shen, Xin Mao, Xinlei Liu, Weining Sun, Wei Yang, Xincheng Ni, Kai Wang, Ling Xu, Zi-Lei Duan, Qing-Cui Zou, Hai-Lin Zhang, Wang Qu, Yang-Hao-Peng Long, Ming-Hua Li, Rui-Cheng Yang, Xiaolong Liu, Jing You, Yangli Zhou, Rui Yao, Wen-Pei Li, Jing-Ming Liu, Pei Chen, Yang Liu, Gui-Feng Lin, Xin Yang, Jun Zou, Linli Li, Yiguo Hu, Guang-Wen Lu, Wei-Min Li, Yu-Quan Wei, Yong-Tang Zheng, Jian Lei, Shengyong Yang

**Affiliations:** 1 State Key Laboratory of Biotherapy, West China Hospital, Sichuan University, Chengdu, Sichuan 610041, China.; 2 Key Laboratory of Animal Models and Human Disease Mechanisms of the Chinese Academy of Sciences, Kunming Institute of Zoology, Chinese Academy of Sciences, Kunming, Yunnan 650223, China.; 3 Kunming National High-level Biosafety Research Center for Non-human Primates, Center for Biosafety Mega-Science, Kunming Institute of Zoology, Chinese Academy of Sciences, Kunming, Yunnan 650107, China.; 4 Key Laboratory of Drug Targeting and Drug Delivery Systems, Ministry of Education, West China School of Pharmacy, Sichuan University, Chengdu, Sichuan 610041, China.; 5 State Key Laboratory of Genetic Resources and Evolution, Kunming Institute of Zoology, Chinese Academy of Sciences, Kunming, Yunnan 650223, China.; 6 National Clinical Research Center for Geriatrics, West China Hospital, Sichuan University, Chengdu, Sichuan 610041, China.

## Abstract

The COVID-19 pandemic caused by severe acute respiratory syndrome coronavirus 2 (SARS-CoV-2) continually poses serious threats to global public health. The main protease (M^pro^) of SARS-CoV-2 plays a central role in viral replication. We designed and synthesized 32 new bicycloproline-containing M^pro^ inhibitors derived from either boceprevir or telaprevir, both of which are approved antivirals. All compounds inhibited SARS-CoV-2 M^pro^ activity in vitro, with 50% inhibitory concentration values ranging from 7.6 to 748.5 nM. The cocrystal structure of M^pro^ in complex with MI-23, one of the most potent compounds, revealed its interaction mode. Two compounds (MI-09 and MI-30) showed excellent antiviral activity in cell-based assays. In a transgenic mouse model of SARS-CoV-2 infection, oral or intraperitoneal treatment with MI-09 or MI-30 significantly reduced lung viral loads and lung lesions. Both also displayed good pharmacokinetic properties and safety in rats.

## Targeting the SARS-CoV-2 main protease

Vaccines are an important tool in the fight against COVID-19, but developing antiviral drugs is also a high priority, especially with the rise of variants that may partially evade vaccines. The viral protein main protease is required for cleaving precursor polyproteins into functional viral proteins. This essential function makes it a key drug target. Qiao *et al.* designed 32 inhibitors based on either boceprevir or telaprevir, both of which are protease inhibitors approved to treat hepatitis C virus. Six compounds protected cells from viral infection with high potency, and two of these were selected for in vivo studies based on pharmokinetic experiments. Both showed strong antiviral activity in a mouse model.

*Science*, this issue p. 1374

## Teaser

Designed SARS-CoV-2 M^pro^ (main protease) inhibitors display excellent antiviral activity both in vitro and in a transgenic mouse model.

The COVID-19 pandemic is caused by severe acute respiratory syndrome coronavirus 2 (SARS-CoV-2) (*1*–*3*). Despite intensive countermeasures implemented around the world, morbidity and mortality remain high with many countries facing a new wave of infections (*4*, *5*). Because limited antiviral agents are available to combat SARS-CoV-2 infection, the development of specific antiviral drugs against SARS-CoV-2 is urgently needed.

SARS-CoV-2 is an enveloped positive-sense single-stranded RNA virus belonging to the genus *Betacoronavirus* (*1*–*3*, *6*). This virus contains a ~30-kb RNA genome encoding two large overlapping polyprotein precursors (pp1a and pp1ab), four structural proteins (spike, envelope, membrane, and nucleocapsid), and several accessory proteins (*1*, *2*, *6*). The cleavage of the two polyproteins (pp1a/pp1ab) into individual nonstructural proteins is essential for viral genome replication. This cleaving process is performed by two viral proteases: main protease (M^pro^, also named 3CL protease) and papain-like protease (*7*). These viral proteases are thus important antiviral targets (*8*, *9*). Notably, M^pro^ exclusively cleaves polypeptides after a glutamine (Gln) residue, and no known human protease displays the same cleavage specificity as M^pro^ (*9*, *10*). This may allow the development of drugs that are specific to M^pro^ to reduce potential side effects.

Despite some SARS-CoV-2 M^pro^ inhibitors being reported (*11*–*21*) and a dipeptidyl inhibitor by Pfizer entering phase I clinical trials (*14*, *15*, *22*), previous literature on inhibitors of SARS-CoV-2 M^pro^ (*11*–*22*) has not included infection data in an animal model. Here, we describe the design of 32 new SARS-CoV-2 M^pro^ inhibitors, two of which show effective antiviral activity in mice.

**Fig. 1:**
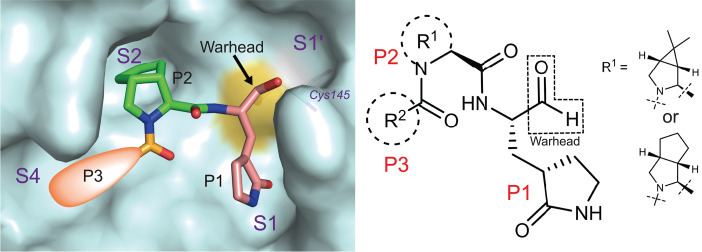
Schematic diagram of the design of novel SARS-CoV-2 M^pro^ inhibitors.

The design of SARS-CoV-2 M^pro^ inhibitors was based on the reported crystal structures of SARS-CoV-2 M^pro^ (*11*–*13*) and our cocrystal structures of SARS-CoV-2 M^pro^ in complex with the approved antivirals against hepatitis C virus infection, boceprevir (PDB entry 7COM) and telaprevir (PDB entry 7C7P) (fig. S1). The active site of M^pro^ is composed of four sites (S1′, S1, S2, and S4), which often accommodate four fragments (P1′, P1, P2, and P3, respectively) of peptidomimetic inhibitors (*8*, *10*). In our design of new inhibitors (Fig. 1), we fixed P1 as an optimal fragment, used P2 that was derived from either boceprevir or telaprevir, and allowed P3 to change. First, an aldehyde was used as the warhead in P1 to form a covalent bond with the catalytic site Cys^145^, which is essential for the antiviral activity (*13*). Relative to other bulky warheads, the small and highly electrophilic aldehyde has been reported to be more potent (*7*, *10*, *20*, *22*). However, the clinical safety of the generated aldehydes remains to be determined because of possible off-target effects due to the high electrophilicity of aldehyde (*23*). Second, we chose a five-membered ring (γ-lactam) derivative of glutamine to occupy the S1 site of M^pro^, which not only mimics the native P1 glutamine of the substrates but also increases the activity of inhibitors (*24*, *25*). Third, we used a bicycloproline moiety, either (1*R*,2*S*,5*S*)-6,6-dimethyl-3-aza-bicyclo[3.1.0]hexane-2-formamide (P2 of boceprevir) or (1*S*,3a*R*,6a*S*)-octahydrocyclopenta[c]pyrrole-1-formamide (P2 of telaprevir), as a P2 fragment. This was inspired by our cocrystal structures of SARS-CoV-2 M^pro^ in complex with boceprevir and telaprevir (fig. S1), in which the two bicycloproline moieties suitably occupy the S2 pocket of M^pro^. In particular, the rigid and hydrophobic bicycloproline is expected to increase drug exposure in vivo (*26*). Finally, by analyzing the characteristics of the S4 site of M^pro^, we decided to use hydrophobic subgroups of medium size for P3 to enhance the potency and pharmacokinetic (PK) properties of the resulting inhibitors. We thus designed and synthesized 32 compounds with various P3 fragments (MI-01 to MI-32; fig. S2). Among these compounds, MI-01 to MI-14 contain P2 of boceprevir, whereas MI-15 to MI-32 include P2 of telaprevir. [See supplementary materials for chemical structures (fig. S2), synthetic routes, and characterization of these compounds by nuclear magnetic resonance and electrospray ionization mass spectrometry.]

The 32 compounds’ biochemical activities against SARS-CoV-2 M^pro^ were determined by a fluorescence resonance energy transfer (FRET) assay. For this, recombinant SARS-CoV-2 M^pro^ protein was prepared. The turnover number (*k*_cat_)/Michaelis constant (*K*_m_) value of the recombinant protein was determined as 50,656 ± 4221 M^–1^ s^–1^, similar to a previous result (*11*). In the FRET assay, all 32 compounds (MI-01 to MI-32) showed potent inhibitory activities on SARS-CoV-2 M^pro^, with 50% inhibitory concentration (IC_50_) values ranging from 7.6 to 748.5 nM (table S1). Of these, 24 compounds displayed two-digit nanomolar IC_50_ values, and three exhibited single-digit values (MI-21, 7.6 nM; MI-23, 7.6 nM; MI-28, 9.2 nM). The positive controls GC376 and 11b, two of the most potent SARS-CoV-2 M^pro^ inhibitors reported (*13*, *17*), exhibited IC_50_ values of 37.4 nM and 27.4 nM in the same assay, respectively. Next, a differential scanning fluorimetry (DSF) assay was performed to validate the direct binding between our compounds and SARS-CoV-2 M^pro^. All the compounds displayed large thermal shifts ranging from 12.5° to 21.7°C (table S1), indicating their tight binding to SARS-CoV-2 M^pro^. It is noteworthy that the two different bicycloproline moieties (P2) did not affect the inhibitory activities and binding abilities (e.g., MI-03 versus MI-21, MI-12 versus MI-28, and MI-14 versus MI-30; table S1 and fig. S2).

**Fig. 2:**
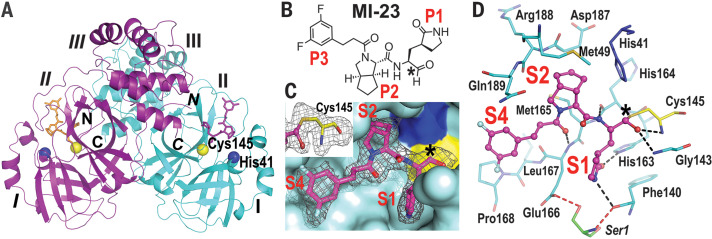
Overall structure of SARS-CoV-2 M^pro^–MI-23 complex. (**A**) Cartoon view of the M^pro^ dimer (molecule A, cyan; molecule B, purple). Three domains (I, II, and III) of each monomer are marked. The catalytic dyad Cys^145^-His^41^ is located in the cleft between domains I and II. MI-23 in both molecules is shown in purple or orange. The N and C termini of each M^pro^ are labeled. Labels for molecule B are in italics. (**B**) The chemical structure of MI-23. (**C**) The MI-23 binding pocket of M^pro^. *F*_o_ – *F*_c_ density map (gray mesh, σ = 2.5) is shown for MI-23 (purple). Cys^145^ and His^41^ are shown in yellow and blue, respectively. The covalent bond is formed by Cys^145^ and the warhead aldehyde. *F*_o_ − *F*_c_ density map (σ = 2.5) is shown in gray. (**D**) Interactions between M^pro^ and MI-23; the hydrogen bonds between them are shown as black dashed lines. Ser^1^ from molecule B interacts with Glu^166^ and Phe^140 ^in molecule A (red dashed lines) to support S1 pocket formation. The warhead carbon is marked with a black asterisk in (B), (C), and (D). Images in (A), (C), and (D) were prepared using PyMOL (https://pymol.org).

To illustrate the detailed binding mode of our compounds with SARS-CoV-2 M^pro^, we determined the 2.0-Å structure of M^pro^ in complex with one of the most active compounds, MI-23 (IC_50_ = 7.6 nM) (Fig. 2, A to D). The crystal structure of the M^pro^–MI-23 complex belongs to space group *C*2 (table S2) with one molecule per asymmetric unit. The biological dimer of M^pro^ is formed by an M^pro^ monomer and its symmetry-mate across the crystallographic two-fold axis (Fig. 2A). MI-23 binds to the active site of M^pro^ as expected (Fig. 2, C and D). The carbon of the warhead aldehyde interacts with the sulfur atom of catalytic residue Cys^145^ to form a 1.8-Å covalent bond (Fig. 2C). The oxygen of the aldehyde forms two hydrogen bonds with the main-chain amides of Cys^145^ and Gly^143^ (forming the “oxyanion hole”) (Fig. 2D). The P1 γ-lactam ring of MI-23 inserts deeply into the S1 pocket. The oxygen and nitrogen of lactam form two hydrogen bonds with the side chain of His^163^ (2.8 Å) and the main chain of Phe^140^ (3.3 Å), respectively. The main-chain amide of P1 makes a 2.9-Å hydrogen bond with the backbone O of His^164^. Because of the conformational restraints inherent in the structure of proline (*27*), the rigid P2 bicycloproline of MI-23 adopts the trans-exo**conformation with restricted N–Cα bond rotation (the ϕ torsion angle is ~ –61.8°). This causes the bicycloproline group to point to the hydrophobic S2 pocket, where it forms hydrophobic interactions with Cγ of Met^165^, Cβ and Cγ of Gln^189^ and His^41^, Cε of Met^49^, and the backbone C and Cα of Asp^187^ and Arg^188^. The backbone oxygen of P3 interacts with the backbone amide of Glu^166^ with a 2.9-Å hydrogen bond. The 1-ethyl-3,5-difluorobenzene moiety of P3 shows an extended conformation and occupies the S4 site. This moiety forms hydrophobic interactions with Cγ of Gln^189^ and the backbone C and Cα of Leu^167^ and Pro^168^ (Fig. 2D). The benzene ring of P3 also forms a very weak hydrophobic interaction with Gly^251^ from an adjacent translational symmetry protomer as a result of crystal packing. Overall, the binding pattern of the representative compound MI-23 with M^pro^ is consistent with our design concept.

We then selected 20 compounds with IC_50_ < 50 nM in the enzyme inhibition assay to examine their cytotoxicity and cellular antiviral activity. First, the cytotoxicity of these compounds was evaluated using the Cell Counting Kit-8 (CCK8) assay (Beyotime Biotechnology), and no compounds showed cytotoxicity [half cytotoxic concentration (CC_50_] > 500 μM] in the cell lines tested, including Vero E6, HPAEpiC, LO2, BEAS-2B, A549, and Huh7 cells (tables S3 and S4).

**Fig. 3:**
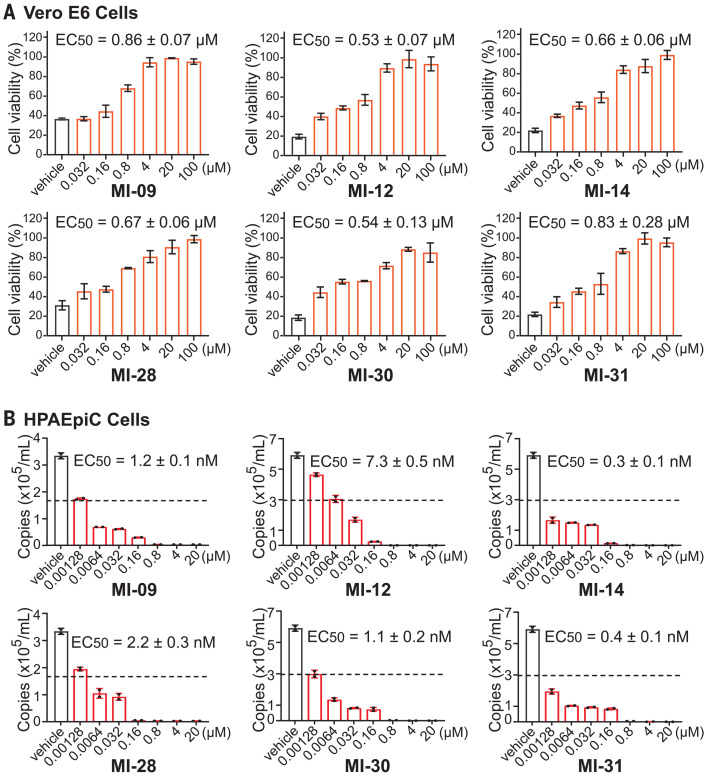
Antiviral activity of six compounds against SARS-CoV-2 in cell-based assays. (**A**) Vero E6 cells were infected with SARS-CoV-2 at a multiplicity of infection (MOI) of 0.1 and treated with different concentrations of test compounds (MI-09, MI-12, MI-14, MI-28, MI-30, and MI-31). At 3 dpi, the cytopathic effect caused by SARS-CoV-2 infection was quantitatively analyzed using CCK8 according to the manufacturer’s protocol. Data are means ± SD; *n* = 3 biological replicates. (**B**) HPAEpiC cells were infected with SARS-CoV-2 at an MOI of 0.01 and treated with different concentrations of test compounds (MI-09, MI-12, MI-14, MI-28, MI-30, and MI-31). At 2 dpi, viral RNA copies (per ml) were quantified from cell culture supernatants by RT-qPCR. Data are means ± SD; *n* = 2 biological replicates.

Next, the compounds’ cellular antiviral activity was examined by a cell protection assay. In this assay, the viability of SARS-CoV-2–infected Vero E6 cells with or without treatment with the compounds was assessed using CCK8. All the compounds dose-dependently protected cells from death with 50% effective concentration (EC_50_) values ranging from 0.53 to 30.49 μM (table S4). Of note, six compounds, including MI-09 (0.86 μM), MI-12 (0.53 μM), MI-14 (0.66 μM), MI-28 (0.67 μM), MI-30 (0.54 μM), and MI-31 (0.83 μM), exhibited nanomolar or low micromolar EC_50_ values (Fig. 3A). We noticed that some compounds (e.g., MI-22 and MI-25) with high potency in the enzymatic assay showed marginal activity in the cell protection assay, perhaps due to relatively low lipophilic groups in P3 and the resulting poor cell membrane permeability (*28*). Quantitative reverse transcription polymerase chain reaction (RT-qPCR) revealed that all six compounds inhibited SARS-CoV-2 virus replication in HPAEpiC cells with low-nanomolar EC_50_ values (0.3 to 7.3 nM) (Fig. 3B). In the same CCK8 and RT-qPCR assays, the positive control GC376 showed EC_50_ values of 1.46 μM and 153.1 nM, respectively, and the corresponding values for 11b were 0.89 μM and 23.7 nM. To further corroborate the antiviral potency of these compounds, we conducted RT-qPCR in another cell line, Huh7. The six compounds showed antiviral EC_50_ values of 31.0 to 96.7 nM, whereas GC376 and 11b displayed EC_50_ values of 174.9 nM and 74.5 nM, respectively (fig. S5).

**Fig. 4:**
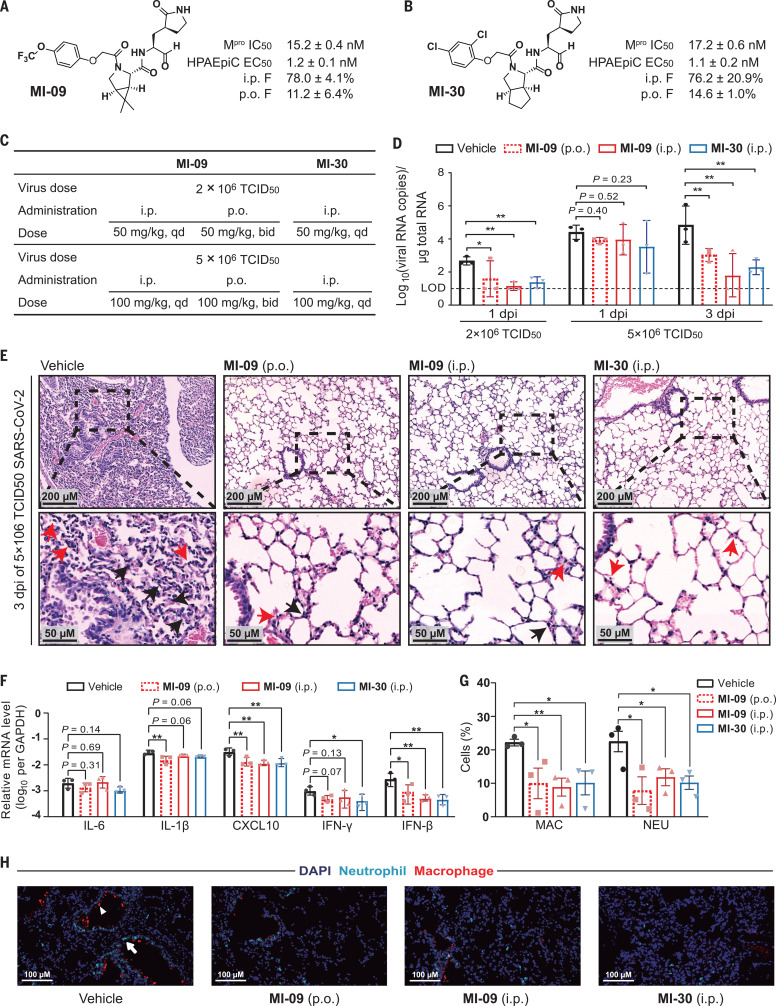
MI-09 and MI-30 reduce lung viral loads and lung lesions in a SARS-CoV-2 infection transgenic mouse model. (**A** and **B**) Chemical structures and summary of in vitro activity data and bioavailability of MI-09 and MI-30. (**C**) Overview of in vivo study design. (**D**) Viral loads in the lungs of SARS-CoV-2–infected hACE2 transgenic mice. Mice infected with the indicated dose of SARS-CoV-2 were treated with MI-09, MI-30, or vehicle solution, and then were killed at 1 or 3 dpi. Five lung lobes of each mouse were collected to determine viral loads. Data (means ± SD) represent the median of five lung lobes of individual mice. The horizontal dotted line shows the viral load limit of detection (LOD) of 1.0 log_10_ RNA copies. Data below the LOD are shown at the LOD. *P < 0.05, **P < 0.01 (two-tailed unpaired Student’s *t* test). (**E**) Representative images of lung histopathological changes from SARS-CoV-2–infected hACE2 mice (5 × 10^6^ TCID_50_) at 3 dpi. Magnified views of the boxed regions for each image are shown below. Black arrows indicate alveolar septal thickening; red arrows point to inflammatory cell infiltration. See fig. S4 for whole-lung tissue scan images of SARS-CoV-2–infected hACE2 mice at 3 dpi. (**F**) Representative chemokine and cytokine assessment of the lung tissues (*n* = 3) of the indicated groups, as detected in lung tissue homogenate at 3 dpi. Data are means ± SD. *P < 0.05, **P < 0.01 versus the vehicle group (one-way analysis of variance). (**G** and **H**) Infiltration analysis for neutrophils and macrophages in the lungs of SARS-CoV-2–infected hACE2 mice (5 × 10^6^ TCID_50_) at 3 dpi. (G) Percentages of macrophages and neutrophils in the lungs. *P < 0.05, **P < 0.01 (unpaired Student’s *t* test). (H) Representative images of fluorescence staining. White triangle and arrow indicate macrophage and neutrophil, respectively.

To identify which of the six compounds is suitable for in vivo antiviral studies, we conducted PK experiments in Sprague-Dawley rats. Two compounds, MI-09 and MI-30, showed relatively good PK properties with oral bioavailability of 11.2% and 14.6%, respectively (table S5). Because a compound with oral bioavailability of >10% has potential for development as an oral drug (*29*), MI-09 and MI-30 were selected for further in vivo antiviral study. The key PK parameters of MI-09 and MI-30 are summarized in Fig. 4, A and B. When administered intravenously (i.v.) (10 mg/kg), intraperitoneally (i.p.) (20 mg/kg), and orally (p.o.) (20 mg/kg), MI-09 showed area under the curve (AUC) values of 7429 hours⋅ng ml^–1^, 11,581 hours⋅ng ml^–1^, and 1665 hours⋅ng ml^–1^, respectively, whereas MI-30 displayed AUC values of 9768 hours⋅ng ml^–1^, 14,878 hours⋅ng ml^–1^, and 2843 hours⋅ng ml^–1^, respectively. After i.p. administration, MI-09 or MI-30 displayed a half-life (*T*_1/2_) of 4.53 hours, a bioavailability of 78.0%, and a clearance rate (CL) of 22.67 ml min^–1^ kg^–1^. The corresponding values for MI-30 were *T*_1/2_ = 3.88 hours, bioavailability = 76.2%, and CL = 17.10 ml min^–1^ kg^–1^. On the basis of the EC_50_/EC_90_ values from HPAEpiC cells, a single i.p. dose of 20 mg kg^–1^ day^–1^ MI-09 or MI-30 maintained the plasma levels at EC_50_ (1.2 nM for MI-09, 1.1 nM for MI-30) and EC_90_ (47.9 nM for MI-09, 58.8 nM for MI-30) for ~24 hours and 6 hours (fig. S3, A and B), respectively. Also, a single p.o. dose of 20 mg kg^–1^ day^–1^ MI-09 or MI-30 sustained the plasma levels at EC_50_ and EC_90_ for ~10 hours and 6 hours (fig. S3, C and D), respectively. Moreover, according to the EC_50_/EC_90_ values from Vero E6 cells, with a single i.p. dose of 20 mg kg^–1^ day^–1^ MI-09 or MI-30, the durations of drug plasma levels above EC_50_ (0.86 μM for MI-09, 0.54 μM for MI-30) and EC_90_ (3.62 μM for MI-09, 2.12 μM for MI-30) were ~3 hours and 2 hours, respectively. A single p.o. dose of 20 mg kg^–1^ day^–1^ MI-09 or MI-30 caused the drug plasma concentrations to reach EC_50_ but not EC_90_ in Vero E6 cells.

MI-09 and MI-30 were then evaluated for their toxicity in rats. In an acute toxicity experiment, no rats died after i.v. (40 mg/kg), i.p. (250 mg/kg), or p.o. (500 mg/kg) treatment with either MI-09 or MI-30 (table S6). In a repeated dose toxicity study, treatment with MI-09 or MI-30 by i.v. at 6 and 18 mg kg^–1^ day^–1^, i.p. at 100 and 200 mg kg^–1^ day^–1^, or p.o. at 100 and 200 mg/kg twice daily for 7 consecutive days did not result in noticeable toxicity in the animals (table S6).

Further, we investigated the in vivo antiviral activity of our compounds in a human angiotensin-converting enzyme 2 (hACE2) transgenic mouse model, which is susceptible to SARS-CoV-2 (*30*). In our pilot study, hACE2 transgenic mice were intranasally inoculated with SARS-CoV-2 [2 × 10^6^ TCID_50_ (50% tissue culture infectious dose) virus per mouse] and were then treated with vehicle (control), MI-09 [50 mg/kg p.o. twice daily (bid) or 50 mg/kg i.p. once daily (qd)] or MI-30 (50 mg/kg i.p. qd) starting at 1 hour prior to virus inoculation (Fig. 4C) and continuing until 5 days post-infection (5 dpi). During the 6-day period, no abnormal behaviors or body weight loss were observed in any animals tested. At 1 dpi, the mean viral RNA loads in the lung tissues of the three treatment groups were significantly (*P* < 0.05, Student’s *t* test) lower than that of the control group (Fig. 4D). At 3 and 5 dpi, the viral RNA loads in the lung tissues of treatment groups were almost undetectable, and those of the control group were also very low [below the limit of detection (LOD)], which might be due to the mild degree of infection.

We thus increased the virus challenge dose of SARS-CoV-2 to 5 × 10^6^ TCID_50_, which mimics a moderate infection. The mice were treated as described above, except that the doses increased to 100 mg/kg for both i.p. and p.o. administration of MI-09 and MI-30 (Fig. 4C). The higher dose of virus challenge led to a higher level of viral loads in the lungs of infected mice, as expected. The mean viral RNA loads in the lung tissues of the three treatment groups were slightly lower than those of the control group at 1 dpi and significantly lower (*P* < 0.05, Student’s *t* test) at 3 dpi (Fig. 4D). At 5 dpi, the viral loads in the lung tissues were undetectable in the treatment groups and were low (near or below LOD) in the control group.

Histopathological analysis was performed for the lungs of mice infected with SARS-CoV-2 at 5 × 10^6^ TCID_50_. At 3 dpi, the vehicle-treated mice showed moderate alveolar septal thickening and inflammatory cell infiltration, whereas all compound-treated animals exhibited slight alveolar septal thickening and mild inflammatory cell infiltration (Fig. 4E). To investigate whether the compounds ameliorate lung damage by affecting host immune response, we studied the expression of inflammatory cytokines and chemokines as well as immune cell infiltration in the lungs. MI-09 or MI-30 reduced the expression levels of IFN-β and CXCL10 (Fig. 4F). Also, fewer neutrophils and macrophages occurred in the lungs of compound-treated mice than in control mice (Fig. 4, G and H), suggesting inhibition of immune cell infiltration. Together, our results show that i.p. or p.o. administration of MI-09 or MI-30 could efficiently inhibit SARS-CoV-2 replication and ameliorate SARS-CoV-2–induced lung lesions in vivo, and they represent an important step toward the development of orally available anti–SARS-CoV-2 drugs.

## Supplementary Materials

science.sciencemag.org/content/371/6536/1374/suppl/DC1 Figs. S1 to S5 Tables S1 to S7 Materials and Methods Supplementary Text References (*31*–*41*) MDAR Reproducibility Checklist
